# Enhancing Heart Transplantation: Utilizing Gas-Loaded Nanocarriers to Mitigate Cold/Hypoxia Stress

**DOI:** 10.3390/ijms25115685

**Published:** 2024-05-23

**Authors:** Chiara Rubeo, Gjylije Hoti, Magalì Giordano, Chiara Molinar, Manuela Aragno, Beatrice Mantuano, Stefano Comità, Saveria Femminò, Roberta Cavalli, Francesco Trotta, Claudia Penna, Pasquale Pagliaro

**Affiliations:** 1 Department of Clinical and Biological Sciences, University of Turin, Regione Gonzole 10, 10043 Orbassano, Italy; chiara.rubeo@unito.it (C.R.); magali.giordano@unito.it (M.G.); manuela.aragno@unito.it (M.A.); beatrice.mantuano@unito.it (B.M.); stefano.comita@unito.it (S.C.); saveria.femmino@unito.it (S.F.); 2 Department of Drug Science and Technology, University of Turin, Via P. Giuria 9, 10125 Torino, Italy; gjylije.hoti@unito.it (G.H.); chiara.molinar@unito.it (C.M.); roberta.cavalli@unito.it (R.C.); 3 Department of Chemistry, University of Turin, Via P. Giuria 7, 10125 Torino, Italy; francesco.trotta@unito.it; 4 National Institute for Cardiovascular Research (INRC), 40126 Bologna, Italy

**Keywords:** cardioplegic solutions, static cold storage, hypoxia/reoxygenation, nanomonomers, nanosponges, molecular signaling pathways

## Abstract

Gas-loaded nanocarriers (G-LN) show promise in improving heart transplantation (HTx) outcomes. Given their success in reducing cell death during normothermic hypoxia/reoxygenation (H/R) in vitro, we tested their integration into cardioplegic solutions and static cold storage (SCS) during simulated HTx. Wistar rat hearts underwent four hours of SCS with four G-LN variants: O_2_- or N_2_-cyclic-nigerosyl-nigerose-nanomonomers (CNN), and O_2_- or N_2_-cyclic-nigerosyl-nigerose-nanosponges (CNN-NS). We monitored physiological-hemodynamic parameters and molecular markers during reperfusion to assess cell damage/protection. Hearts treated with nanomonomers (N_2_-CNN or O_2_-CNN) showed improvements in left ventricular developed pressure (LVDP) and a trend towards faster recovery of the rate pressure product (RPP) compared to controls. However, nanosponges (N_2_-CNN-NS or O_2_-CNN-NS) did not show similar improvements. None of the groups exhibited an increase in diastolic left ventricular pressure (contracture index) during reperfusion. Redox markers and apoptosis/autophagy pathways indicated an increase in Beclin 1 for O_2_-CNN and in p22phox for N_2_-CNN, suggesting alterations in autophagy and the redox environment during late reperfusion, which might explain the gradual decline in heart performance. The study highlights the potential of nanomonomers to improve early cardiac performance and mitigate cold/H/R-induced stunning in HTx. These early improvements suggest a promising avenue for increasing HTx success. Nevertheless, further research and optimization are needed before clinical application.

## 1. Introduction

Heart transplantation (HTx) stands as a critical and historical lifeline, offering a vital solution for severe cardiac conditions in patients facing refractory heart failure [1,2]. Challenges, including the narrow window for optimal organ viability during transportation and the heart’s vulnerability to hypoxia/reoxygenation (H/R), have impeded the full success of HTx. While static cold storage (SCS) continues to be the predominant method, its use comes with a significant risk associated with prolonged cold ischemic periods. The standard procedure, involving hypothermic cardioplegic solutions, shows some limitations, thereby driving investigations into novel strategies for mitigating H/R damage during transplantation. Despite the growing interest in machine perfusion systems, ongoing debates persist regarding their superiority over SCS [3]. This study focuses on addressing the overarching issue of hypoxia-related complications in HTx. Specifically, early post-reperfusion stunning can significantly jeopardize the success of HTx [4].

Previous studies explored oxygen gas-loaded nanocarriers (G-LN) in vitro, specifically focusing on α-cyclodextrin-based nanosponges as oxygen carriers in reducing cell mortality during normothermic-H/R, regardless of whether they were applied before or after hypoxia [5]. Additionally, the use of O_2_-cyclic-nigerosyl-nigerose (O_2_-CNN) for controlled oxygen delivery demonstrated a 15–30% reduction in cell mortality during H/R [6]. These studies highlighted the potential applications of G-LN in preventing normothermic ischemia/reperfusion injury (IRI). Accordingly, lipid nanoparticles also play a significant role in controlling the release and diffusion of gases within biological tissues in gas-transmitter applications [7]. Whether these G-LN may be protective in hypothermic conditions remains to be elucidated.

Acknowledging SCS as the predominant technique in HTx field, our study sought to investigate the possible benefits of G-LN in enhancing SCS outcomes. Given the ongoing debate on the safety and efficacy of oxygen therapy in IRI contexts [8], our proposed solution involves integrating G-LN with and without oxygen into both the cardioplegic solution and SCS. Leveraging the anti-inflammatory properties of nanocarriers [9], our study is pioneering in investigating the protective effects of G-LN in hypothermic settings.

Thus, our objectives were to explore the functional and molecular changes induced by SCS, evaluate whether the integration of G-LN could effectively mitigate cold/H/R injury resulting from SCS, and assess whether G-LN could enhance organ preservation and improve heart performance upon reperfusion. To address these objectives, we utilized Wistar rats whose hearts were buffer-perfused. Following a stabilization period, cardiac arrest was induced using the National Institutes of Health (NIH) cardioplegic solution, either enriched with G-LN or not. Specifically, we compared two types of G-LN with different kinetics of gas release, namely nanocarriers (CNN) and nanosponges (CNN-NS) [10]. Accordingly, we utilized four types of G-LN for comparison: fully oxygen-charged variants, designated as O_2_-CNN-nanomonomers (O_2_-CNN) and O_2_-CNN-nanosponges (O_2_-CNN-NS), alongside their fully nitrogen-charged counterparts (N_2_-CNN and N_2_-CNN-NS). Subsequently, the hearts underwent SCS in G-LN-enriched NIH solution prior to reperfusion. Physiological parameters such as left ventricular developed pressure (LVDP) and heart rate (HR) were monitored before and after SCS, while molecular analyses were conducted to assess changes in molecular and redox markers associated with H/R-induced damage.

## 2. Results

### 2.1. Characteristics of Cardioplegic Solutions Containing CNN or CNN-NS

The characteristics of NIH containing CNN or CNN-NS are reported in Table 1.

The presence of CNN and CNN-NS, either oxygen-loaded or unloaded, produced a slight increase in the pH of the NIH solution, but the values are still suitable for the hearth SCS. Moreover, this increase in pH is in line with the temperature dependency of the ionic product of water: the ionic balance shifts towards a higher pH at lower temperatures and vice versa [11,12,13].

CNN and CNN-NS formulations showed the capability to store oxygen, releasing the gas upon dilution with prolonged kinetics confirming the previous results [10]. In particular, CNN significantly contributed to extending the duration of oxygen release in the NIH solution, as it has the capability to trap oxygen within the molecule’s inner cavity. This prolonged oxygen release is evidenced by the higher O_2_ concentration observed at 4 °C compared to the notable decrease in gas concentration in NIH after 6 h. Remarkably, even after 24 h, the O_2_ concentration remains elevated. Interestingly, the ability of CNN to sustain O_2_ release was further enhanced by the inclusion of CNN-NS. The differences between CNN-NS and CNN in NIH are highlighted by a slower and more controlled release achieved through CNN-NS, particularly within the initial 6 h of recorded oxygen release. The entrapment of oxygen is also noticeable at 37 °C, emphasizing the critical role played by the polymer network within the matrix and the cavities. No hemolytic activity was observed after the incubation of the two formulations with red blood cells [10].

### 2.2. Contractile Recovery of Hearts Arrested and Preserved with CNN

**Figure 1:**
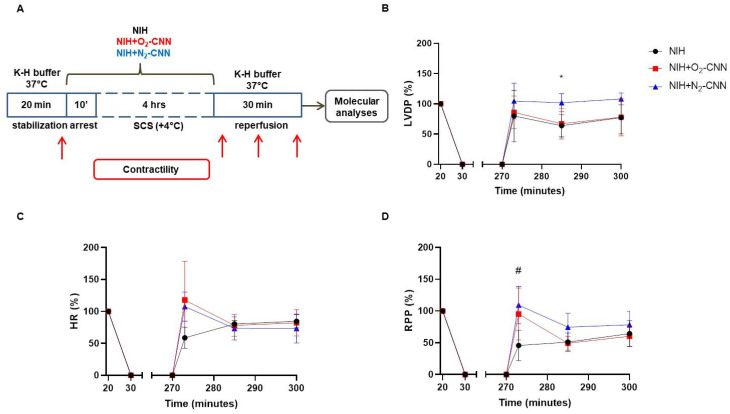
Experimental protocol and hemodynamic parameters of rat hearts arrested and preserved for 4 h in NIH supplemented or not with O_2_- or N_2_-CNN. (**A**) Diagram of the experimental protocol. Red arrows indicate the moments when LVDP and HR were recorded. Measurements of (**B**) LVDP, (**C**) HR and (**D**) RPP, expressed as percentage of the basal level. Data are shown as mean ± SD, n = 4–5, * adjusted *p* = 0.0422 NIH + N_2_-CNN vs. NIH; # adjusted *p* = 0.0393 NIH + N_2_-CNN vs. NIH.

To assess whether CNN charged with O_2_ or N_2_ could enhance heart contractile recovery post-cardioplegia, we replicated heart transplantation phases ex vivo using the Langendorff system (Figure 1, panel A). LVDP at the end of stabilization served as the baseline. Hearts were included in the analysis when they developed more than 55 mmHg (LVDP = sLVP − dLVP). Following stabilization, hearts were perfused with NIH, with or without CNN enrichment, prompting rapid heart arrest. After 4 h of cold storage at 4 °C in the same NIH, hearts underwent 30 min of reperfusion, during which HR and LVP were monitored (Figure 1, panel A).

In NIH hearts (black lines in Figure 1; panels B–D), LVDP was 65.47 ± 3.11 mmHg at baseline. LVDP recovered by about 70% following SCS during both early and later reperfusion phases. HR gradually regained vigor throughout reperfusion, approaching nearly 80% of the baseline by the end of the reperfusion period. RPP demonstrated a continuous increase during reperfusion, reaching about 65% of the baseline level by the end.

In NIH + N_2_-CNN hearts (blue lines in Figure 1; panels B–D), LVDP returned to baseline or even surpassed it early in reperfusion, maintaining a high level throughout, reaching 108.1 ± 9.9% of the baseline LVDP after 30 min of reperfusion. Of note, LVDP was significantly higher compared to the NIH group (101.7 ± 14.8% vs. 63.7 ± 18.5%, adjusted *p* = 0.0422; in the middle of reperfusion). HR in this group surged early but declined steadily thereafter, with RPP exhibiting a similar trend. Importantly, this NIH + N_2_-CNN group showed significantly higher RPP than the NIH group during early reperfusion (109.2 ± 29.4% vs. 45.8 ± 23.8%, adjusted *p* = 0.0393).

In NIH + O_2_-CNN hearts (red lines in Figure 1; panels B–D), LVDP recovery mirrored that of NIH hearts. HR surged during early reperfusion but declined steadily during middle and late reperfusion stages, with RPP following a similar pattern. Notably, there was no statistically significant difference in RPP between NIH + N_2_-CNN and NIH + O_2_-CNN hearts.

### 2.3. Contracture Index in Hearts Arrested and Preserved with CNN

In cases of necrosis, dLVP, an indicator of contracture, typically increases [14,15]. In hearts treated solely with NIH, the dLVP at stabilization measured 1.30 ± 1.78 mmHg, remaining similar at the conclusion of reperfusion, at 5.63 ± 7.71 mmHg (*p* = N.S.). Also, in hearts treated with CNN, the dLVP at stabilization and at the end of reperfusion did not demonstrate any significant difference between each other. This was observed both with NIH + N_2_-CNN (2.61 ± 5.23 mmHg at baseline vs. 11.15 ± 4.61 mmHg at the end of reperfusion; *p* = N.S.) and with NIH + O_2_-CNN (8.03 ± 7.48 mmHg at baseline vs. 4.76 ± 7.60 mmHg at the end of reperfusion; *p* = N.S.).

### 2.4. Contractile Recovery of Hearts Arrested and Preserved with CNN-NS

**Figure 2:**
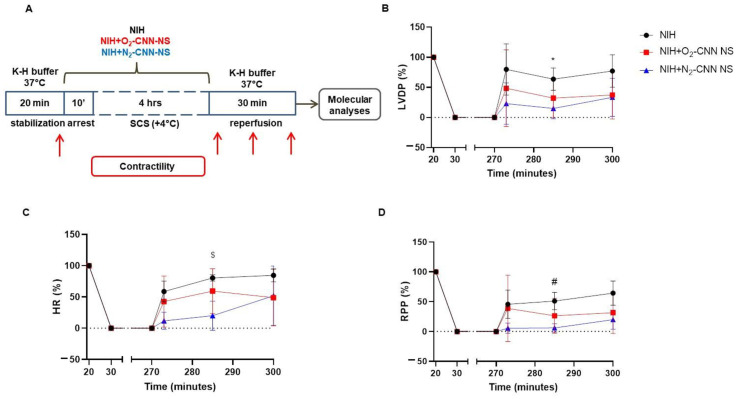
Experimental protocol and hemodynamic parameters of rat hearts arrested and preserved for 4 h in NIH supplemented or not with O_2_- or N_2_-CNN-NS. (**A**) Diagram of the experimental protocol. Red arrows indicate the moments when LVDP and HR were recorded. Measurements of (**B**) LVDP, (**C**) HR and (**D**) RPP, expressed as percentage of the basal level. Data are shown as mean ± SD, n = 4–5, * adjusted *p* = 0.0386 NIH vs. NIH + N_2_-CNN-NS; # adjusted *p* = 0.0162 NIH vs. NIH + N_2_-CNN-NS; $ adjusted *p* = 0.0124 NIH vs. NIH + N_2_-CNN-NS.

Following the experimental procedures outlined in the preceding section, we employed CNN-NS charged with either O_2_ or N_2_ (Figure 2). In both the NIH + N_2_-CNN-NS (Figure 2, blue lines) and NIH + O_2_-CNN-NS groups (Figure 2, red lines), the recovery of LVDP, HR, and RPP after 4 h of storage showed no significant difference compared to the NIH group. Of note, statistical significance was only observed for the N_2_-CNN-NS group during the middle phase of reperfusion for all three parameters when compared to the NIH group (LVDP 15.0 ± 17.4% vs. 63.7 ± 18.5%, adjusted *p* = 0.0386; HR 20.1 ± 23.3 vs. 80.3 ± 5.2%, adjusted *p* = 0.0124; RPP 6.0 ± 7.0% vs. 51.0 ± 14.5%, adjusted *p* = 0.0162).

### 2.5. Contracture Index in Hearts Arrested and Preserved with CNN-NS

In hearts treated with CNN-NS as well, the dLVP exhibited no significant difference between stabilization and the end of reperfusion in any group (NIH + O_2_-CNN-NS: 5.81 ± 7.31 mmHg vs. 3.37 ± 5.13 mmHg, *p* = N.S.; NIH + N_2_-CNN-NS: 4.65 ± 4.57 mmHg vs. 12.00 ± 8.93 mmHg, *p* = N.S.).

### 2.6. Levels of Apoptosis Indices in Hearts Treated with Different Nanocarriers

As contracture was absent, our focus shifted to programmed cell death rather than necrosis. To assess the impact of the NIH solution, with and without G-LN, on key signaling pathways implicated in IRI and hypoxia response, we conducted Western blots on myocardial samples obtained at the end of reperfusion.

**Figure 3:**
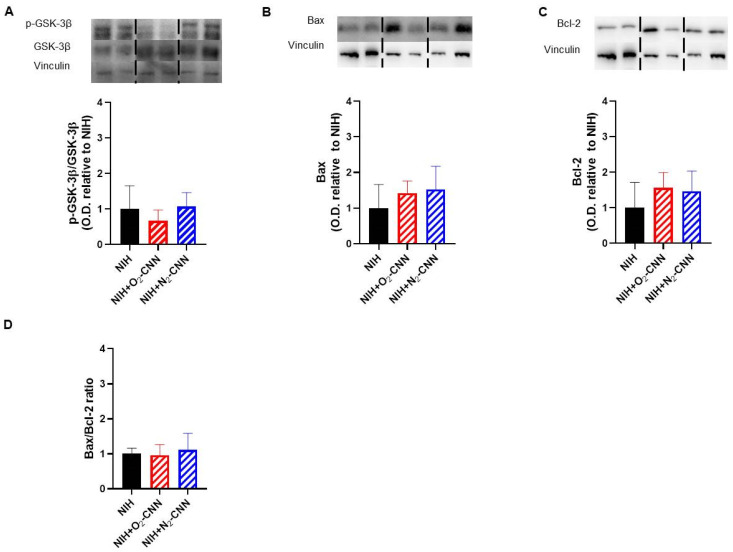
Levels of apoptosis indices in hearts treated with CNN. Representative blots and quantified results of p-GSK-3β normalized to total GSK-3β protein levels (**A**), Bax (**B**), Bcl-2 (**C**), ratio between Bax and Bcl-2 (**D**). Anti-vinculin antibodies were used for the loading control. Values are shown as mean ± SD of optic density (O.D.) and are expressed as folds to mean NIH. n = 4–5. Blots were selectively cropped to display only pertinent representative images (vertical dotted lines). The original gels and blots are available for viewing in the Supplementary Materials.

**Figure 4:**
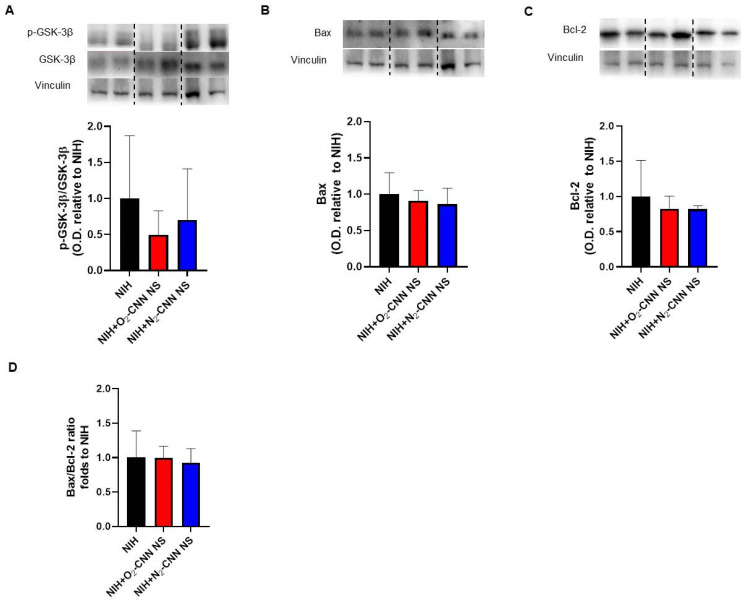
Levels of apoptosis indices in hearts treated with CNN-NS. Representative blots and quantified results of p-GSK-3β normalized to total GSK-3β protein levels (**A**), Bax (**B**), Bcl-2 (**C**), ratio between Bax and Bcl-2 (**D**). Anti-vinculin antibodies were used for the loading control. Values are shown as mean ± SD of O.D. and are ex-pressed as folds to mean NIH. n = 4–5. Blots were selectively cropped to display only pertinent representative images (vertical dotted lines). The original gels and blots are available for viewing in the Supplementary Materials.

Figure 3 displays the apoptosis indices for hearts stored with CNN, while Figure 4 depicts those stored with CNN-NS. Notably, all analyzed molecules, including GSK-3β phosphorylation, Bax, Bcl-2, and the Bax/Bcl-2 ratio, exhibited similar levels among hearts treated with NIH supplemented with CNN or CNN-NS compared to those without supplementation.

### 2.7. Levels of Autophagy and Oxidative Stress Indices in Hearts Treated with Different Nanocarriers

**Figure 5:**
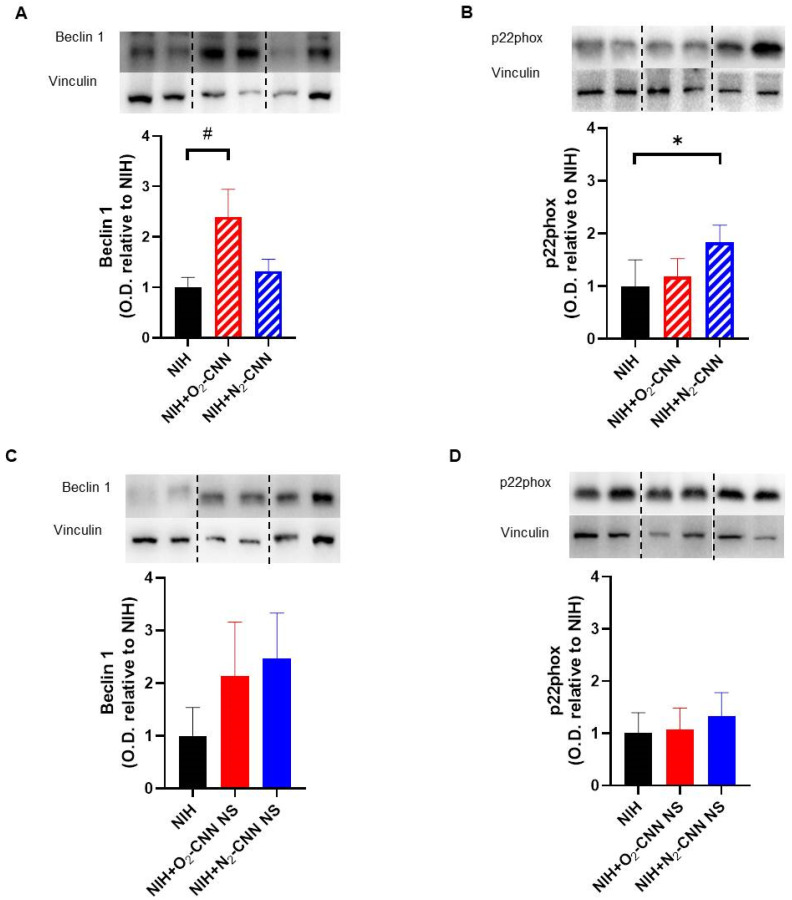
Levels of autophagy and oxidative stress in hearts treated with different nanocarriers in the cardioplegic solution. Quantified results of Beclin 1 (**A**) and p22phox protein levels in hearts treated with CNN (**B**). Quantified results of Beclin 1 (**C**) and p22phox protein levels in hearts treated with CNN-NS (**D**). Anti-vinculin antibodies were used for the loading control. Values are shown as mean ± SD of O.D. and are expressed as folds to NIH. n = 4–5. * Adjusted *p* = 0.0372; # adjusted *p* = 0.0102. Blots were selectively cropped to display only pertinent representative images (vertical dotted lines). The original gels and blots are available for viewing in the Supplementary Materials.

Panel A of Figure 5 illustrates the autophagy index (Beclin 1) and panel B depicts the oxidative stress marker (p22phox) for hearts stored with CNN. Remarkably, Beclin 1 levels were markedly higher in hearts treated with O_2_-CNN compared to those stored in NIH alone (almost 2.5 times higher, adjusted *p* = 0.0102). Conversely, p22phox, a constituent of NADPH oxidase, exhibited a significant increase in hearts treated with N_2_-CNN compared to NIH-treated hearts (approximately 1.8 times higher, adjusted *p* = 0.0372).

Panels C and D of Figure 5 present the corresponding data for hearts stored with CNN-NS. Importantly, there were no significant variations observed in either Beclin 1 (adjusted *p* = 0.0646 NIH + N_2_-CNN vs. NIH) or p22phox levels in hearts stored with CNN-NS compared to those stored solely in the NIH solution.

### 2.8. Catalase Activity in Hearts Stored with NIH and CNN

**Figure 6:**
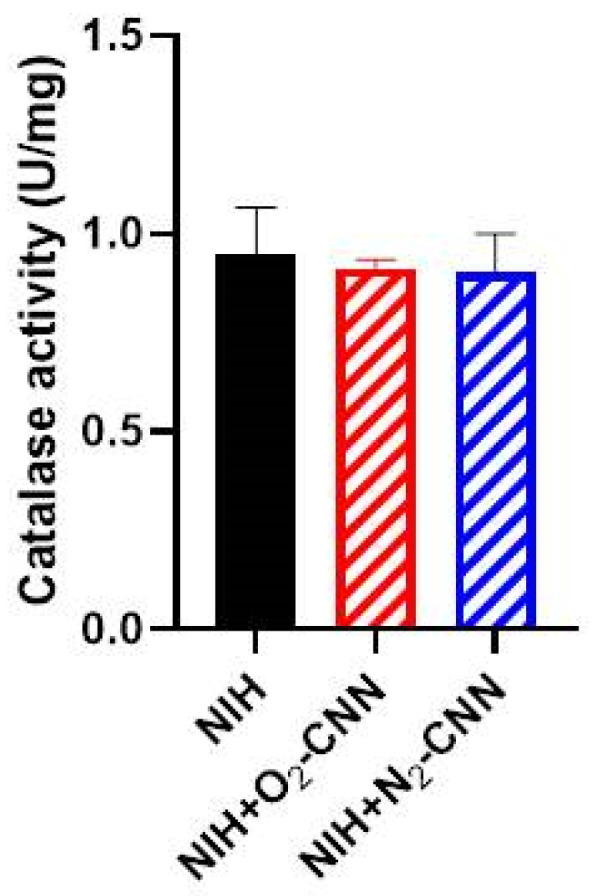
Levels of catalase activity. The graph shows the levels of catalase activity in hearts treated with CNN. Data are mean ± SD. n = 3–4.

Considering the observed improvements in heart performance and the increase in p22phox levels in hearts stored with NIH + N_2_-CNN, we conducted further investigations into catalase activity. This analysis involved measuring catalase activity in lysates obtained from hearts perfused and stored with NIH, both with and without CNN. The findings revealed a substantial level of enzyme activity, with no significant differences observed among the three groups (NIH = 0.95 ± 0.24 U/mg tissue; NIH + O_2_-CNN = 0.91 ± 0.04 U/mg tissue; NIH + N_2_-CNN = 0.90 ± 0.17 U/mg tissue) (Figure 6).

## 3. Discussion

Severe cardiac conditions often necessitate HTx, yet challenges persist in achieving optimal organ viability. SCS preserves organ viability by temporally halting cellular metabolism, enabling successful transplantation. Its simplicity and affordability contribute to improved transplant outcomes and facilitate equitable organ distribution. Ongoing research aims to address limitations, such as cold ischemia injury, to further enhance SCS effectiveness in organ preservation. In our study, we aimed to address these challenges by integrating G-LN (O_2_-CNN, N_2_-CNN, O_2_-CNN-NS, or N_2_-CNN-NS) into NIH and SCS.

Our exploration of G-LN integration stemmed from their promising therapeutic applications in various medical contexts [9,16,17]. To simulate heart transplantation, we initially investigated the functional and molecular changes induced by SCS. The absence of contracture in hearts treated solely with cardioplegic solution, as well as those supplemented with G-LN, provides valuable insights. Indeed, ischemic contracture, or “stone heart,” is a condition that can hinder heart transplantability. Our findings suggest that specific G-LN inclusion effectively mitigated cold/H/R injury associated with SCS (e.g., myocardial stunning), thereby improving organ preservation. The transiently enhanced heart performance observed in hearts treated with CNN further highlights the therapeutic potential of this approach. This indicates the need for further exploration of the mechanisms underlying G-LN supplementation’s protective effects, potentially offering novel strategies to optimize organ preservation during SCS.

### 3.1. Nanomonomers Transiently Increase Heart Performance Recovery

We revealed a significant initial enhancement in RPP and cardiac performance in hearts preserved with CNN, highlighting an important therapeutic opportunity for accelerated recovery within a delicate clinical framework. This underscores the potential for timely interventions by physicians who have the possibility to effectively bolster the heart after transplantation surgery, likely limiting myocardial stunning and leading to further optimizations and advancements in cardiac function.

Specifically, cardioplegic solutions enriched with CNN, particularly those charged with nitrogen, proved effective in enhancing early contractile recovery. Nevertheless, the improvement was temporary, as no discernible differences among the groups were observed by the end of reperfusion. This decline in contractile force towards the end of reperfusion could be attributed to an increase in either autophagy (increase in Beclin 1) or redox stress, as evidenced by elevated catalase activity and/or levels of p22phox. The latter indicates heightened NADPH oxidase activity, an enzyme complex involved in generating anion superoxide (O_2_-) within cells [18]. Importantly, dLVP does not exhibit an increase in all groups following SCS and reperfusion, suggesting that this procedure offers protection against necrosis. Indeed, it is well-established that in the event of necrosis, diastolic pressure would typically rise [14,15]. Hence, we ruled out for the role of necrosis and our focus shifted towards markers of programmed cell death, specifically apoptosis and autophagy.

While the O_2_-CNN-charged variant does not yield a significant improvement compared to the NIH solution alone, there is no statistical distinction in the transient beneficial effect induced by N_2_-CNN compared to O_2_-CNN. The difference in performance observed when CNN are enriched with nitrogen or oxygen may be sustained by a different mechanism: in hearts treated with O_2_-CNN, at the end of reperfusion, we observed an increase in Beclin-1, which may suggest an upregulation of autophagy in response to cellular stressors, such as oxidative stress and nutrient deprivation [19,20]. Yet, in N_2_-CNN, at the end of reperfusion, we observe an increase in oxidative stress as revealed by p22phox. In the myocardium, antioxidant proteins like catalase, glutathione peroxidase, and peroxiredoxin work to neutralize H_2_O_2_, with catalase being the primary contributor, as it accounts for roughly 80% of peroxidase activity in cardiomyocytes [21]. At the end of reperfusion, the catalase activity is high in all groups studied, suggesting that further enhancement is not possible when redox stress increases, even in hearts treated with N_2_-CNN, where p22phox is upregulated.

An increase in Beclin 1 was observed in all hearts studied with the exception of those exposed to the CNN charged with nitrogen. This observation, together with the robust level of catalase activity, supports the hypothesis that SCS and subsequent reperfusion induce a remarkable oxidative stress. After the transient improvement, the decline in force may be due to either autophagy or to further redox stress. While we have a hypothesis supported by data explaining the decline in contraction force, we lack a clear explanation for the initial transient improvement. This improvement is likely attributed to reduced stunning but requires further investigation using diverse nanodevices with varying kinetics of gas release.

We would emphasize that although no differences were observed at the end of the reperfusion period, the initial improvement in heart performance may represent a potential window for better recovery in a clinical setting, when an adequate support of the heart activity may further improve the performance [22,23].

### 3.2. Nanosponges Do Not Improve Heart Performance Recovery

Hearts treated with N_2_-CNN-NS or O_2_-CNN-NS demonstrated slightly lower LVDP and RPP, indicating a non-beneficial impact on heart function. The precise reasons for the observed transitory negative impact on heart performance after SCS with CNN-NS remain to be determined, warranting further investigation.

We can speculate that the differences between O_2_-CNN and O_2_-CNN-NS may arise from variations in the kinetics of oxygen release at different temperatures [10]. However, hemodynamic differences were observed mainly between the two types of nanocarriers employed (nanocarriers vs. nanosponges) rather than between the two gases used for their loading (oxygen or nitrogen). Thus, the addition of oxygen in an already protected setting, such as that achieved through cardioplegia and low temperature, seems ineffective both when heart performance is enhanced by CNN and when it is slightly impaired by CNN-NS, at least for the canonical 4 h of SCS. Therefore, while the differing kinetics of gas release [10] may partially explain variations in heart performance, the type of gas is not the determining factor, as the primary distinctions are found among the different types of nanodevices.

### 3.3. Gas-Loaded Nanocarriers: Influence on Redox Stress, Apoptosis, and Autophagy

The modified expression profiles in redox stress, apoptosis, and autophagy signaling pathways underscore the intricate interplay among distinct G-LN formulations and their impact on cellular processes. Specifically, CNN have shown to exert transient cardioprotective effects as revealed by transient improvement in hemodynamics. However, increased autophagic activity (evidenced by elevated Beclin 1 expression) or elevated levels of the oxidative stress marker p22phox may explain the subsequent decline in heart performance. Indeed, autophagy and redox stress are double-edged swords that can transition from being beneficial to detrimental factors [19,20,24,25]. Further exploration into the time-course of these cellular processes is necessary. The present study confirms evidence against a relevant role of apoptosis in this context [14,15].

### 3.4. Methodological Considerations

Monomeric compounds offer promising prospects for enhancing heart performance during prolonged SCS, especially when natural oxygen reserves diminish significantly. It is well known that the reduction in temperature to 4 °C, combined with chemical cardiac arrest, can reduce myocardial oxygen consumption by up to 97%. Consequently, relying solely on oxygen binding to myoglobin may suffice for a limited duration (i.e., 3–4 h) [26], underscoring the necessity for oxygen release to sustain more prolonged cold storage. The application of G-LN in simulated HTx, particularly within the context of SCS, underscores its potential as a novel strategy. However, the subtle effects observed with different G-LN formulations highlight the necessity for further investigation to optimize their application in clinical settings. Although the distinct kinetics of the two nanodevices utilized exposed the heart to varying gas concentrations, testing different concentrations of O_2_ and N_2_ might also be considered. However, this presents a challenge as nanocarriers must be fully charged with a gas to eliminate other atmospheric gases, including atmospheric O_2_ and N_2_. Moreover, nanocarriers release charged gas based on temperature variations [10]. In our study, to simulate clinical conditions, heart storage requires a constant temperature of 4 °C, while reperfusion necessitates 37 °C. Consequently, only distinct nanodevices may facilitate varying gas releases and concentrations, which could be explored in future research. While our data reveal a transient improvement in heart performance upon reperfusion, partially attributable to limitations of stunning, they do not offer a comprehensive understanding of the mechanisms underlying CNN’s enhancement of heart recovery. Elevated levels of serum troponin T have been associated with acute rejection rather than ischemia/reperfusion necrosis during cold storage [4]. Hence, in our model, which excludes organ rejection and contracture, our focus was on analyzing heart recovery and molecular changes signaling in programmed cell death rather than necrosis. Finally, a limitation of our study is the exclusive use of male rats, which restricts the generalizability of our findings. Female rats may exhibit different physiological responses [27]. Future studies should include both sexes to ensure broader applicability of the results.

## 4. Materials and Methods

### 4.1. Preparation of Nanomonomers and Nanosponges

Native CNN and CNN-NS have been previously described [10]. In brief, CNN is a natural non-reducing carbohydrate in which four glucopyranose units are bound by alternating α-1,6 and α-1,3 glucosidic linkages. CNN was cross-linked with carbonyldiimidazole to obtain CNN-NS as previously reported [28]. CNN-NS are solid nanoporous nanoparticles with sizes of about 200 nm formed by a cluster of CNN. Aqueous formulations were prepared by dissolving CNN in NIH solution or dispersing CNN-NS in NIH solution. NIH solution (mmol/L: Na^+^ 98.9, Cl^−^ 107.8, K^+^ 30.0, Ca^++^ 1.0, HCO_3_^−^ 22.0, Glucose 152.6, Mannitol 68.6) was obtained according to the literature recipe [10]. To prepare gas-loaded samples, the NIH formulations were saturated with an oxygen or nitrogen purge at a flux of 4 L/min under stirring, while the gas concentration was monitored up to 35 mg/L in the external aqueous phase. Hemolysis assay was employed for the evaluation of the biocompatibility of the two formulations.

### 4.2. Animals and Ethics Statement

Ten-to-twelve-week-old male Wistar rats (Envigo, Italy) were maintained in accordance with the European Directive 2010/63/EU on the protection of animals used for scientific purposes. The animal protocols implemented in this study received approval from the Ethics Committee of the University of Turin (No. E669c.n.kuz). Following one week of quarantine under standard conditions (25 °C, with a regular light–dark cycle, and access to food and water ad libitum), rats were anesthetized with isoflurane and euthanized by guillotine.

### 4.3. Heart Perfusion

Hearts were swiftly excised and immersed in an ice-cold Krebs–Henseleit (K-H) buffer composed of 130 NaCl, 5.6 KCl, 2.2 CaCl_2_, 0.6 MgCl_2_, 1.4 NaH_2_PO_4_, 25 NaHCO_3_, and 12.2 glucose (in mM). Following this, the hearts were cannulated via the aorta and connected to a Langendorff apparatus for retrograde perfusion with a warm K-H buffer maintained at a constant temperature of 37 °C. The buffer, enriched with a mixture of 95% O_2_ and 5% CO_2_, remained at 37 °C throughout both the stabilization and reperfusion phases. A small incision was made in the left ventricular (LV) wall to facilitate proper drainage of the thebesian flow. Additionally, a homemade polyvinyl-chloride balloon was inserted into the LV and connected to an electro-manometer to record LV pressure (LVP). Saline was used to fill the balloon and achieve an end-diastolic LVP ranging from 5 to 10 mm Hg. Coronary perfusion pressure (CPP) was then adjusted to 80 mmHg to achieve a coronary flow (CF) of 9 ± 1 mL/min·g. Continuous monitoring of CPP, CF, and LVP ensured the stability of the experimental setup.

Following a 20 min stabilization period, hearts were perfused for 10 min with NIH supplemented or not with G-LN. Consequently, five different groups of arrested hearts were studied:

- NIH only (NIH, n = 5);
- NIH plus CNN carrying O_2_ (NIH + O_2_-CNN, n = 4);
- NIH plus CNN-NS carrying O_2_ (NIH + O_2_-CNN-NS, n = 5);
- NIH plus CNN carrying N_2_ (NIH + N_2_-CNN, n = 4);
- NIH plus CNN-NS carrying N_2_ (NIH + O_2_-CNN-NS, n = 4).

The arrested hearts were stored at 4 °C for 4 h in the same NIH with or without G-LN and then reperfused for 30 min with warm K-H buffer (Figure 1A).

Physiological parameters, including systolic left ventricular pressure (sLVP), diastolic left ventricular pressure (dLVP), left ventricular developed pressure (LVDP = sLVP − dLVP), heart rate (HR), and the rate pressure product (RPP; LVDP (mmHg) × HR (bpm)), were monitored and recorded using LabChart v8.1.25 software (National Instruments, Milano, Italy).

Data analysis was conducted during stabilization and reperfusion phases. Recordings were manually analyzed by a blinded investigator who selected a period during which at least 6 beats were free from arrhythmias.

### 4.4. Western Blots

Following the 30 min reperfusion period, rat heart samples were promptly snap-frozen. Cardiac samples were homogenized in RIPA lysis buffer supplemented with protease and phosphatase inhibitors (1:1000, Merck, Darmstadt, Germany). After homogenization and centrifugation, supernatants were subjected to sodium dodecyl sulfate–polyacrylamide gel electrophoresis, transferred onto nitrocellulose membranes (GVS Filter Technology, Bologna, Italy), and blocked in 10% non-fat milk at room temperature for 1 h. Primary antibodies against p-GSK-3β (Santa Cruz Biotechnology, Dallas, TX, USA, sc-11757-R), GSK-3β (Santa Cruz Biotechnology, sc-9166), Bax (Cell Signaling Technology, Danvers, MA, USA, #2772), Bcl-2 (Cell Signaling Technology, #2870), p22phox (Cell Signaling Technology, Danvers, MA, USA, #88048), and Beclin 1 (Abclonal, Woburn, MA, USA, A21191) were incubated with the membranes overnight at 4 °C. The next day, the membranes were exposed to HRP-conjugated anti-rabbit or anti-mouse secondary antibodies (Cell Signaling Technology, Danvers, MA, USA) diluted 1:10,000 for 1 h at room temperature. Membranes were developed using an enhanced chemiluminescence (ECL) detection system (Bio-Rad, Hercules, CA, USA). Images were captured with the ChemiDoc™ XRS+ System (Bio-Rad, Hercules, CA, USA), and Bio-Rad Image Lab Software 6.0.1 was utilized to analyze the immunoreactive bands. Anti-vinculin (Santa Cruz Biotechnology, Dallas, TX, USA) served as the loading control.

### 4.5. Catalase Activity Assay

Catalase activity was measured by fluorescence in heart tissue lysates according to kit instructions (Catalase Activity Assay Kit, Abcam, Cambridge, UK). Catalase activity was determined by the rate of H_2_O_2_ consumption in the samples by the enzyme and expressed as units of catalase activity per milligram of tissue.

### 4.6. Statistical Analysis

Data are expressed as mean ± standard deviation (SD). Calculations were performed using Graphpad Prism 8. Normality of data distribution was assessed by Shapiro–Wilk test. Differences between groups were analyzed by one-way analysis of variance followed by Bonferroni test for multiple comparisons and by Mann–Whitney test. The statistically significant level was set at adjusted *p* < 0.05. “N.S.” stands for “not statistically significant”.

## 5. Conclusions

This study provides valuable insights into the potential benefits of CNN in improving the performance of heart transplantation in early reperfusion. Indeed, CNN transiently improved hemodynamic parameters by about 50% during early reperfusion. Moreover, the possibility for loading these nanodevices with cardioprotective drugs further enhances their therapeutic versatility [29]. By effectively improving hemodynamic associated with reperfusion after cold hypoxia, CNN emerge as a promising avenue for advancing organ preservation and mitigating cold-storage-induced injury. The incorporation of these nanomonomers within a cardioplegic solution may have a positive effect on early reperfusion cardiac performance after transplantation. These encouraging findings establish a solid groundwork for subsequent research and future clinical studies aimed at prolonging the benefits and optimizing heart transplantation practices.

## Figures and Tables

**Table 1:** pH and viscosity values of the cardioplegic formulations at 4 °C.

	pH ± SD	Viscosity ± SD (mpa)
NIH	7.93 ± 0.01	1.194 ± 0.001
NIH + O_2_-CNN	8.07 ± 0.04	1.204 ± 0.004
NIH + O_2_-CNN-NS	8.37 ± 0.01	1.190 ± 0.001
NIH + N_2_-CNN	8.20 ± 0.02	1.204 ± 0.04
NIH + N_2_-CNN-NS	8.42 ± 0.01	1.195 ± 0.001

## Data Availability

The authors confirm that the data supporting the findings of this study are available within the article and after reasonable request.

## Supplementary Materials

The following supporting information can be downloaded at: https://www.mdpi.com/article/10.3390/ijms25115685/s1.
