# Supplementary material for: Enhancing Heart Transplantation: Utilizing Gas-Loaded Nanocarriers to Mitigate Cold/Hypoxia Stress
Source: Int J Mol Sci. 2024 May 23;25(11):5685. doi: 10.3390/ijms25115685 (PMC11171608; doi:10.3390/ijms25115685)
Supplement: Supplementary file 1 [file ijms-25-05685-s001.zip › ijms-3008167-supplementary.pdf]

Original blots (asterisks indicate the bands selected for the representative blots)

Figure 3 A

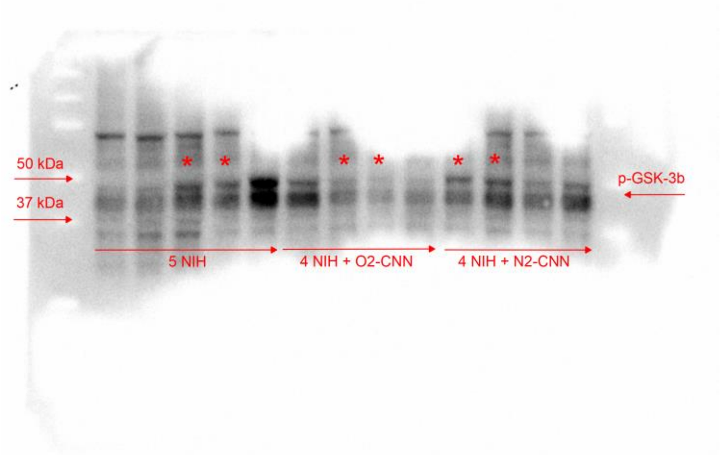

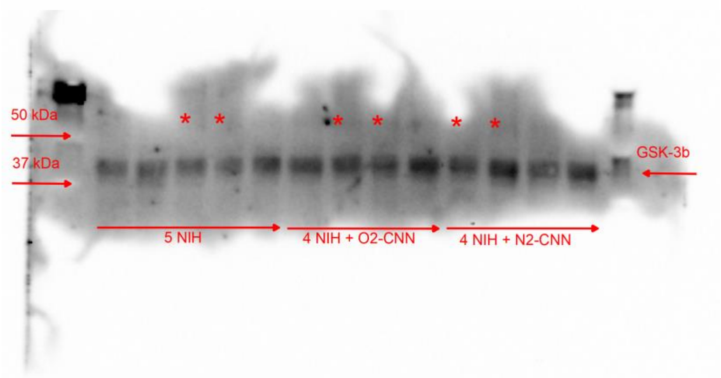

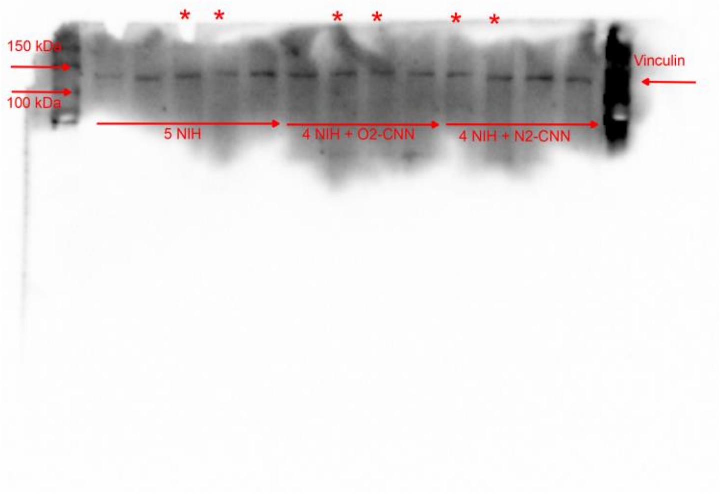

Figure 3 B

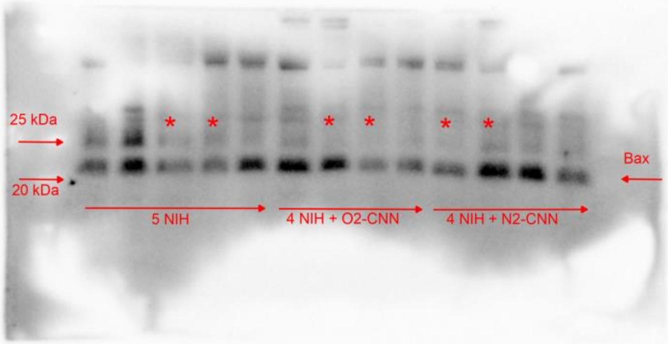

25 kDa
20 kDa
Bax
5 NIH
4 NIH + O2-CNN
4 NIH + N2-CNN

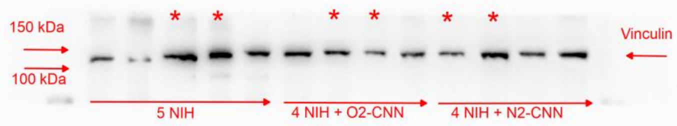

150 kDa
100 kDa
Vinculin
5 NIH
4 NIH + O2-CNN
4 NIH + N2-CNN

Figure 3 C

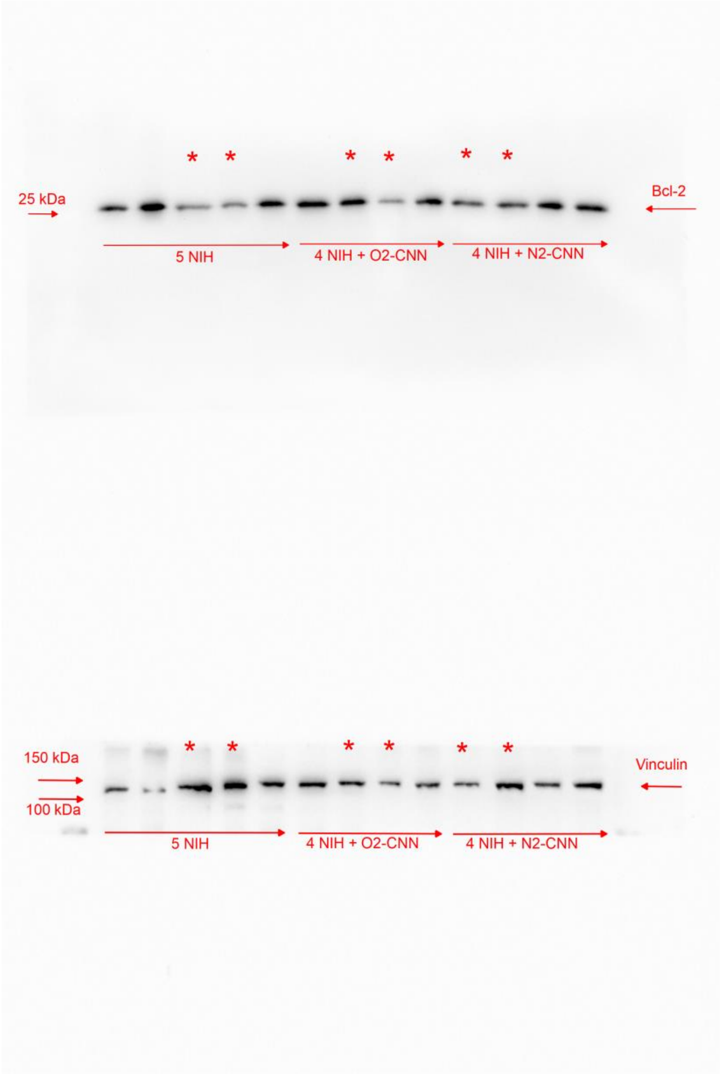

25 kDa
Bcl-2
5 NIH
4 NIH + O2-CNN
4 NIH + N2-CNN
150 kDa
100 kDa
Vinculin
5 NIH
4 NIH + O2-CNN
4 NIH + N2-CNN

Figure 4 A

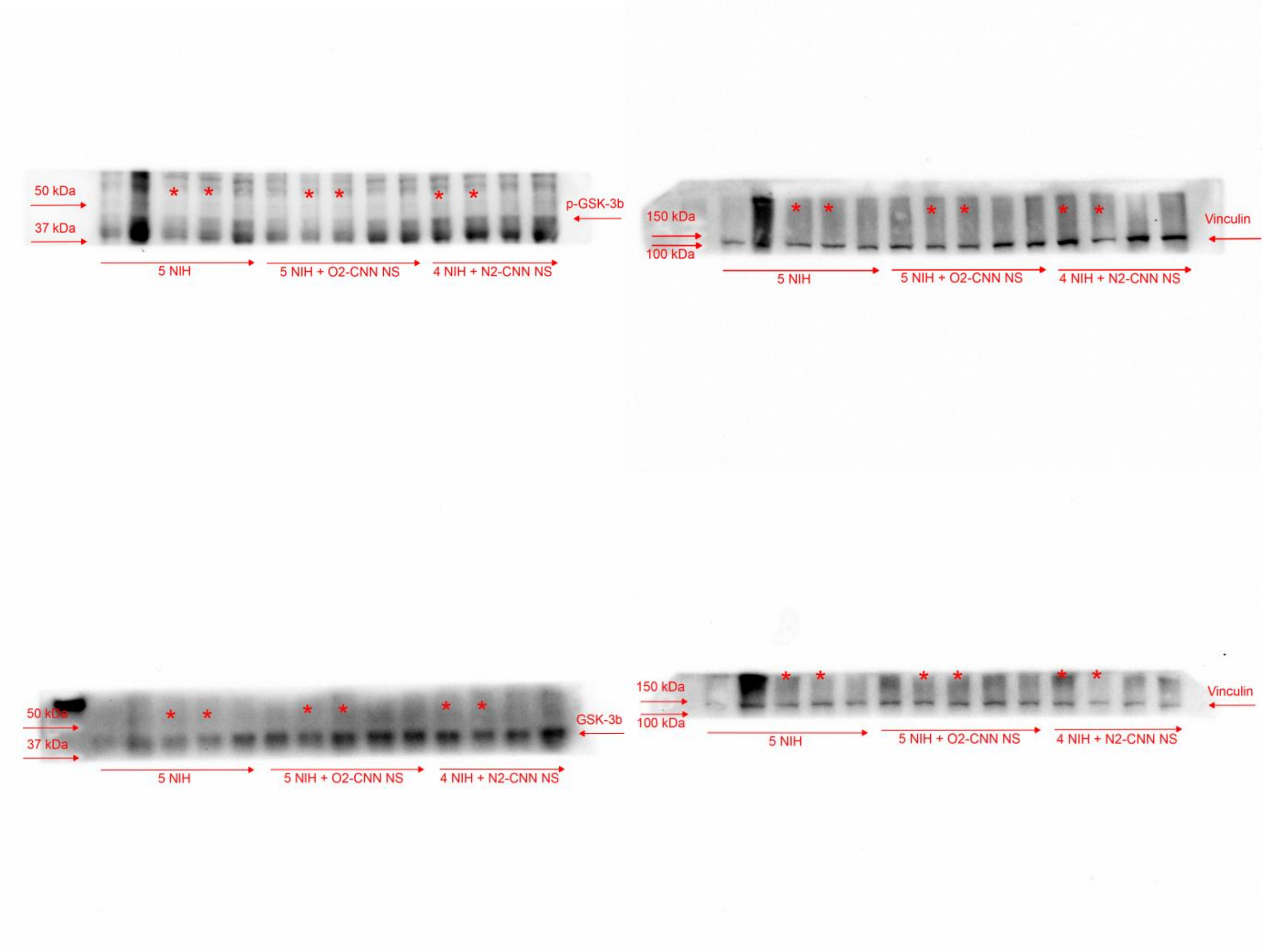
50 kDa
37 kDa
p-GSK-3b
5 NIH
5 NIH + O2-CNN NS
4 NIH + N2-CNN NS
150 kDa
100 kDa
Vinculin
5 NIH
5 NIH + O2-CNN NS
4 NIH + N2-CNN NS
50 kDa
37 kDa
GSK-3b
5 NIH
5 NIH + O2-CNN NS
4 NIH + N2-CNN NS
150 kDa
100 kDa
Vinculin
5 NIH
5 NIH + O2-CNN NS
4 NIH + N2-CNN NS

Figure 4 B

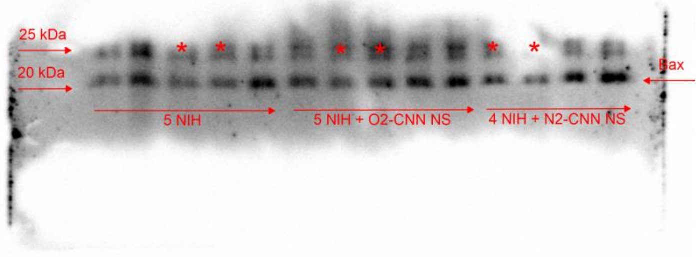
25 kDa
20 kDa
Bax
5 NIH
5 NIH + O2-CNN NS
4 NIH + N2-CNN NS

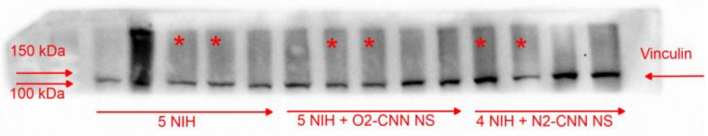
150 kDa
100 kDa
Vinculin
5 NIH
5 NIH + O2-CNN NS
4 NIH + N2-CNN NS

Figure 4 C

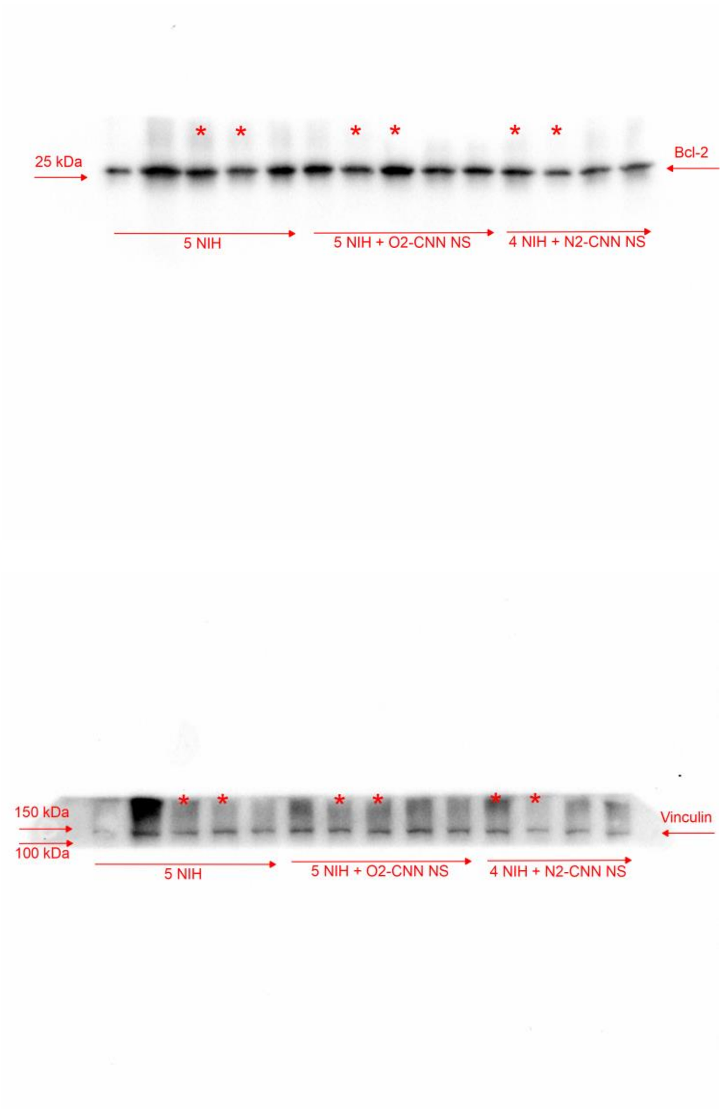

25 kDa
Bcl-2
5 NIH
5 NIH + O2-CNN NS
4 NIH + N2-CNN NS
150 kDa
100 kDa
Vinculin
5 NIH
5 NIH + O2-CNN NS
4 NIH + N2-CNN NS

Figure 5 A

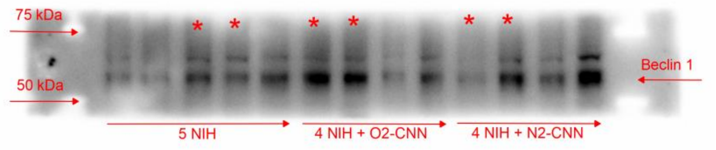
75 kDa
50 kDa
Beclin 1
5 NIH
4 NIH + O2-CNN
4 NIH + N2-CNN

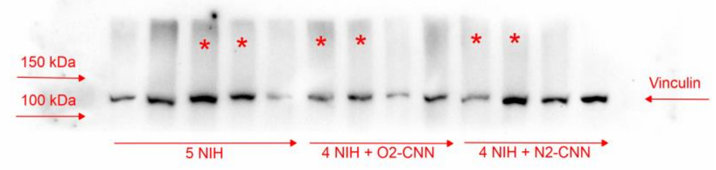
150 kDa
100 kDa
Vinculin
5 NIH
4 NIH + O2-CNN
4 NIH + N2-CNN

Figure 5 B

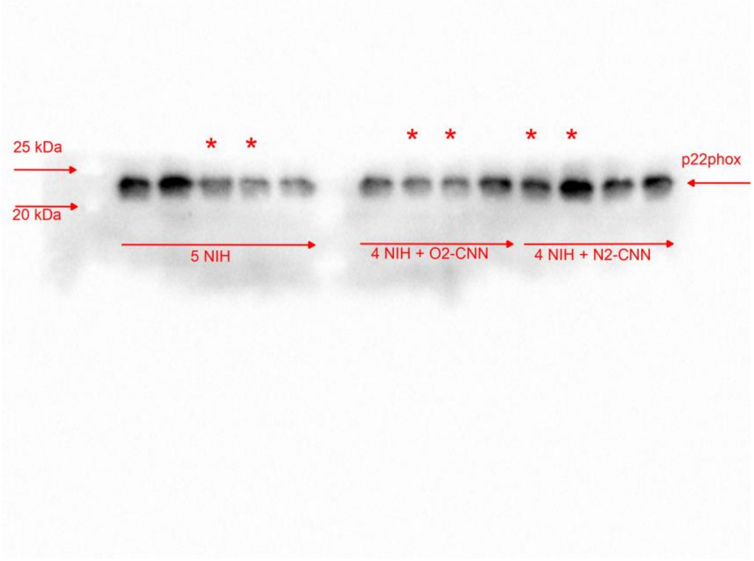
25 kDa
20 kDa
p22phox
5 NIH
4 NIH + O2-CNN
4 NIH + N2-CNN

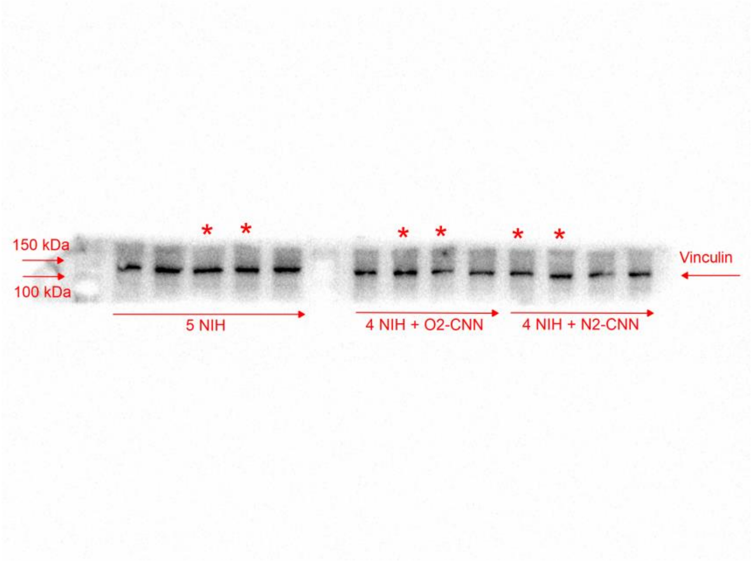
150 kDa
100 kDa
Vinculin
5 NIH
4 NIH + O2-CNN
4 NIH + N2-CNN

Figure 5 C

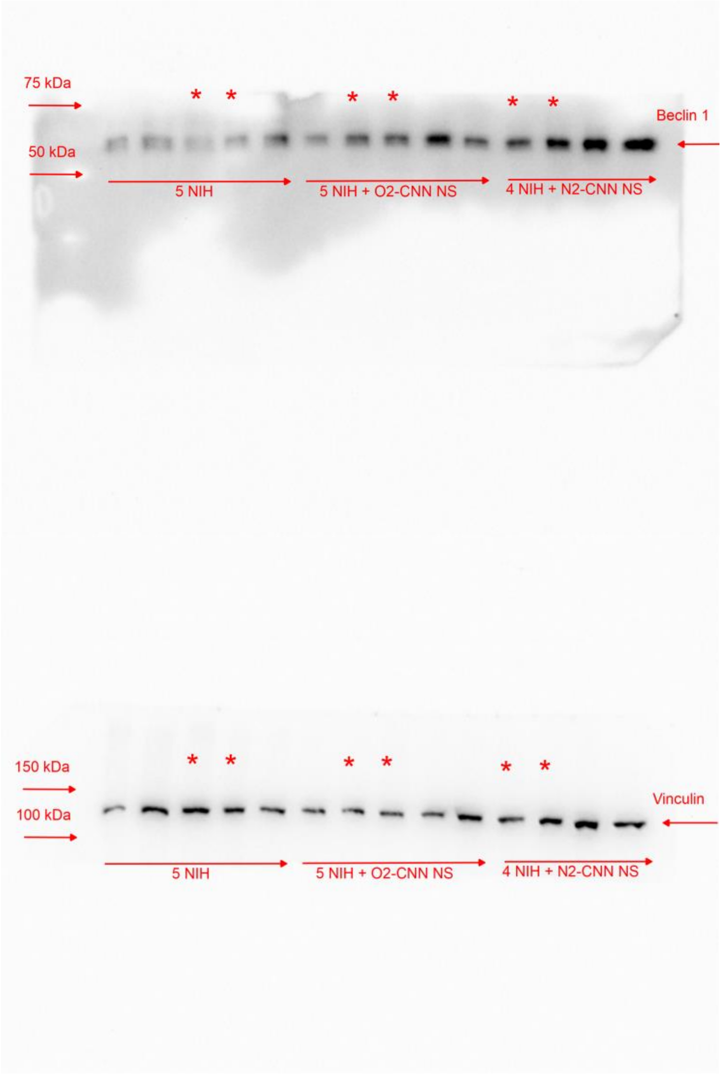
75 kDa
50 kDa
* *
* *
* *
Beclin 1
5 NIH
5 NIH + O2-CNN NS
4 NIH + N2-CNN NS
150 kDa
100 kDa
Vinculin
5 NIH
5 NIH + O2-CNN NS
4 NIH + N2-CNN NS

Figure 5 D

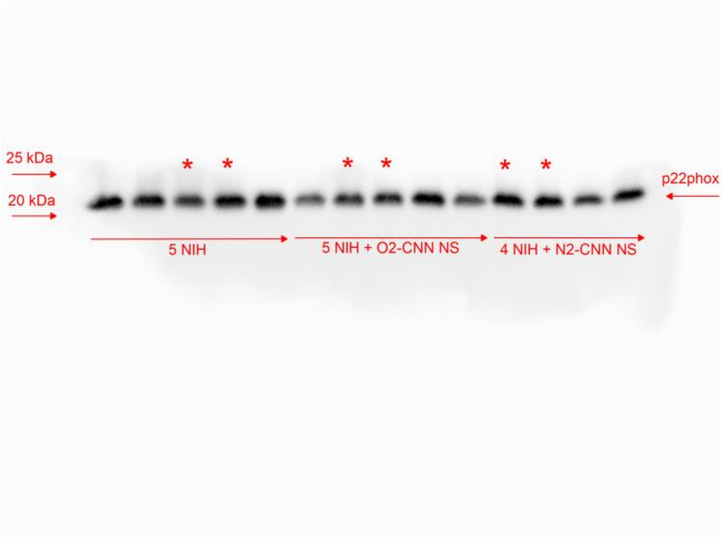

25 kDa
20 kDa
* * * * * *
p22phox
5 NIH
5 NIH + O2-CNN NS
4 NIH + N2-CNN NS

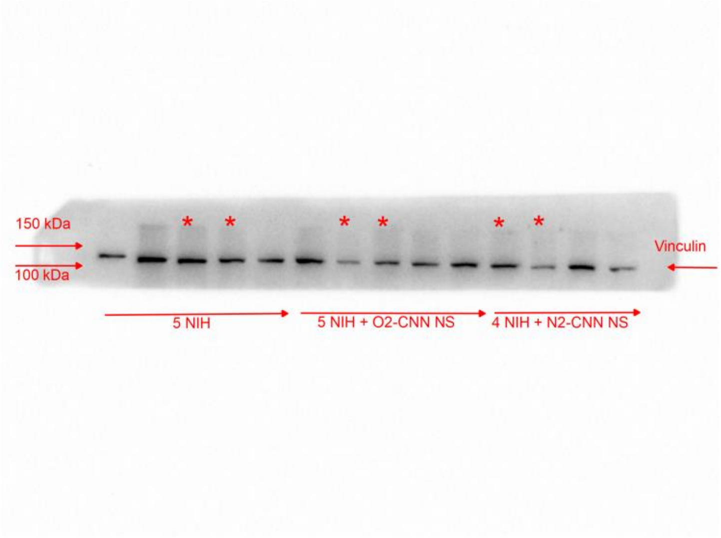

150 kDa
100 kDa
* * * * * *
Vinculin
5 NIH
5 NIH + O2-CNN NS
4 NIH + N2-CNN NS
